# Rituximab, Apremilast, and Upadacitinib as Selected Biosimilar and Targeted Synthetic Disease-Modifying Antirheumatic Drugs with Diverse Mechanisms of Action: Their Current Use in Slowing Down the Progression of Disease

**DOI:** 10.3390/jcm14082605

**Published:** 2025-04-10

**Authors:** Piotr Kawczak, Igor Jarosław Feszak, Tomasz Bączek

**Affiliations:** 1 Department of Pharmaceutical Chemistry, Faculty of Pharmacy, Medical University of Gdańsk, 80-416 Gdańsk, Poland; tomasz.baczek@gumed.edu.pl; 2 Institute of Health Sciences, Pomeranian University in Słupsk, 76-200 Słupsk, Poland; igorfeszak@gmail.com; 3 Department of Nursing and Medical Rescue, Institute of Health Sciences, Pomeranian University in Słupsk, 76-200 Słupsk, Poland

**Keywords:** inflammatory arthritis, targeted treatment, bsDMARDS, tsDMARDS, PDE4i, JAK1i

## Abstract

**Background/Objectives**: Inflammatory arthritides includes a range of joint disorders, such as osteoarthritis and rheumatoid arthritis, as well as inflammatory conditions like gout and lupus. This review investigates the pathophysiology, therapeutic challenges, and evolving treatment landscape of arthritis, with a particular focus on the clinical roles of rituximab, apremilast, and upadacitinib. **Methods**: A comprehensive analysis was undertaken to evaluate the current clinical application, therapeutic efficacy, and safety profiles of selected biosimilar and targeted synthetic disease-modifying antirheumatic drugs (bsDMARDs and tsDMARDs). This overview placed particular emphasis on three key agents—rituximab, apremilast, and upadacitinib—each exemplifying distinct immunomodulatory mechanisms. By focusing on these agents, the analysis highlights the evolving landscape of targeted therapies in rheumatology and underscores the importance of personalized treatment selection based on the disease phenotype, prior therapeutic responses, and comorbid conditions. **Results**: Rituximab, apremilast, and upadacitinib each present valuable therapeutic options for patients who have shown inadequate response to conventional disease-modifying antirheumatic drugs (DMARDs) or nonsteroidal anti-inflammatory drugs (NSAIDs). **Conclusions**: Despite the complexity and heterogeneity of arthritis, agents like rituximab, apremilast, and upadacitinib have expanded the therapeutic possibilities in treating this disease and improved its management. Continued research is essential to optimize patient-specific treatment strategies and explore novel molecular targets.

## 1. Introduction

Arthritis refers to a group of disorders characterized by inflammation, stiffness, and degeneration of the joints. It encompasses a wide range of conditions, including degenerative diseases such as osteoarthritis and immune-mediated forms like rheumatoid arthritis (RA) and psoriatic arthritis (PsA). Inflammatory types of arthritis also include gout, systemic lupus erythematosus (SLE), and septic arthritis, each of which is associated with unique pathophysiological mechanisms and clinical manifestations [1]. Rheumatoid arthritis is a chronic, systemic autoimmune disorder in which the immune system mistakenly attacks the body’s own tissues, particularly the synovial membrane lining the joints. This results in persistent synovitis, cartilage degradation, and bone erosion. The disease is driven by a complex interplay of genetic predisposition and environmental triggers, leading to the production of autoantibodies—such as rheumatoid factor (RF) and anti-citrullinated protein antibodies (ACPAs)—that target self-proteins and promote chronic inflammation [2,3].

Psoriatic arthritis, another significant inflammatory joint disease, occurs in individuals with psoriasis and is characterized by both joint and skin involvement. Unlike RA, PsA may present with asymmetric joint inflammation, enthesitis, dactylitis, and axial skeleton involvement. It is considered a seronegative spondyloarthropathy and is associated with immune dysregulation that involves pathways such as the IL-17, IL-23, and TNF-α pathways. PsA affects up to 30% of people with psoriasis and can lead to progressive joint damage and disability if left untreated. Both RA and PsA have systemic consequences that extend beyond joint involvement. In RA, the chronic inflammatory state contributes to an increased risk of cardiovascular disease, interstitial lung disease, and neurological complications. Similarly, PsA is associated with metabolic syndrome, insulin resistance, and an elevated risk of cardiovascular morbidity. In both conditions, comorbidities such as type 2 diabetes, ischemic heart disease, and chronic obstructive pulmonary disease (COPD) are common and further elevate the risk of premature mortality [4,5,6]. These complexities underscore the importance of early diagnosis, risk stratification, and personalized, multidisciplinary approaches to the management of arthritis.

At the tissue level, inflammation in the synovial membrane leads to the infiltration and activation of immune cells (e.g., T and B cells, neutrophils, macrophages, fibroblasts), leading to the release of growth factors, chemokines, and cytokines that drive chronic inflammation and subsequent cartilage and bone degradation [7]. This persistent inflammatory state elevates cardiovascular risks, with women facing a two- to three-fold increased risk of myocardial infarction even without traditional risk factors, highlighting the need for comprehensive cardiovascular management [8,9]. Additionally, excessive pro-inflammatory cytokine production disrupts bone remodeling, contributing to osteoporosis [10].

Early diagnosis is crucial, and autoantibodies serve as key biomarkers for improved diagnosis, prognosis, and treatment decisions regarding arthritis. Other factors—such as epigenetic changes, post-translational modifications, and T-cell activity—further influence the disease’s progression [11]. RA is also marked by a thickened synovial membrane, the presence of autoantibodies like rheumatoid factor (RF) and anti-citrullinated protein antibodies (ACPAs), and the activation of pathways, e.g., mitogen-activated protein kinases (MAPKs) driven by reactive oxygen species (ROS), all of which lead to irreversible joint and tissue damage. Although current treatments (DMARDs, NSAIDs, corticosteroids) are effective, their long-term use is limited by significant side effects [12,13]. Recent studies also point to a role for m6A modification in RA development [14].

Although a wide spectrum of therapeutic options is available—including analgesics, corticosteroids, and biologic response modifiers—many patients continue to experience inadequate symptom control or disease progression. This therapeutic limitation is largely attributed to the intricate and multifaceted nature of rheumatoid arthritis (RA), which involves disruptions in immune regulation and apoptotic pathways, such as aberrant programmed cell death [15]. Emerging evidence suggests that specific immune cell populations, including memory B cells and invariant natural killer T (iNKT) cells, play pivotal roles in initiating and perpetuating the inflammatory processes that are characteristic of RA. The inconsistent clinical efficacy observed across commonly used therapies—such as conventional DMARDs, glucocorticoids, NSAIDs, and cytokine-targeted biologics—highlights the heterogeneity of disease mechanisms observed among patients. This variability emphasizes the urgent need for more detailed insights into the cellular and molecular underpinnings of RA, which could inform the development of more precise, mechanism-based therapeutic strategies [16,17,18].

The European Alliance of Associations for Rheumatology has defined difficult-to-treat (D2T) RA as cases where at least two different biologic or targeted synthetic DMARDs have failed, often due to additional factors like comorbidities, obesity, and fibromyalgia. These guidelines stress confirming the inflammatory pathology before altering the treatment plans [19]. Ongoing research into new DMARDs and combination therapies, along with advances in targeted drug design, promises more effective treatments. Nonetheless, while DMARDs have reduced the disease activity for many, some patients remain unresponsive, highlighting the need for novel therapeutic targets and multidisciplinary management approaches—including early pharmacological intervention, physical and occupational therapy, and patient education [20,21,22].

## 2. Biosimilar Disease-Modifying Antirheumatic Drugs (bsDMARDs)

BsDMARDs enhance patient access to more affordable biotechnological treatments. Biosimilars offer a cost-effective alternative to reference biologics, improving access to targeted therapies. These drugs closely resemble approved biologics, and their formulations are approaching equivalent efficacy and safety. While preclinical studies have established their similarity, full confirmation requires clinical trials that assess their pharmaco-kinetics, efficacy, safety, and immunogenicity. Unlike small-molecule drugs, biologics have complex structures, making the development of biosimilars challenging due to their inherent variability. Ensuring consistency is key to their effectiveness [23].

### Anti-CD20–Mediated B-Cell Depletion Agent—Rituximab

**Figure 1:**
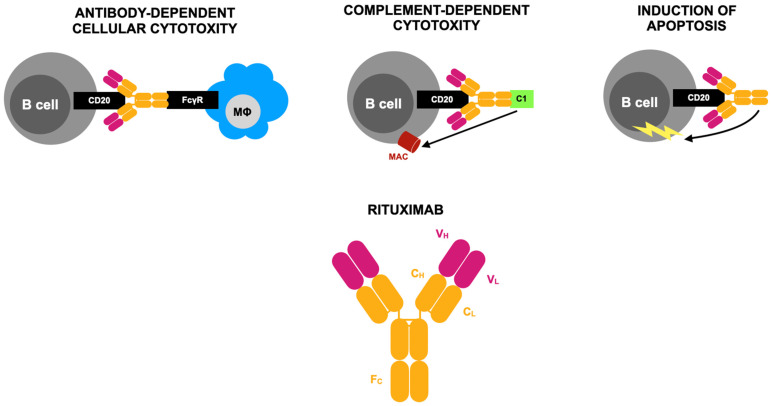
Mode of action of rituximab according to [29]. CD20, a surface protein found on both immature and mature B cells, serves as the target for a chimeric monoclonal antibody composed of both human and mouse components. This antibody specifically binds to the human CD20 antigen. Its structure includes variable regions from a murine anti-CD20 antibody consisting of heavy (VH) and light (VL) chains that form the antigen-binding site. These regions are connected to the constant light (CL) and heavy (CH) regions, along with the Fc portion of human IgG1. B-cell depletion occurs through three primary mechanisms. First, the Fc domain interacts with phagocytic cells, including macrophages, via the Fc gamma receptor (FcγR), which recognizes the carboxy-terminal constant region of the IgG molecule. This interaction triggers antibody-dependent cellular cytotoxicity. Second, binding to the CD20 antigen can activate the complement system, leading to the assembly of membrane attack complexes (MACs) that ultimately cause complement-dependent cell lysis. Third, programmed cell death (apoptosis) is induced. Activation of the complement system is initiated by the C1 protein complex, which consists of C1q, C1r, and C1s subunits. This complex plays a crucial role in triggering the classical complement activation pathway.

Rituximab (RTX), a chimeric anti-CD20 monoclonal antibody, is used to treat B-cell non-Hodgkin lymphomas, chronic lymphoid leukemia (CLL), multiple sclerosis (MS), and autoimmune diseases like rheumatoid arthritis (RA). It depletes B cells through direct cytotoxicity, complement-dependent cytotoxicity (CDC), antibody-dependent cellular cytotoxicity (ADCC), and phagocytosis (ADCP) via Fcγ receptors on immune cells. The mechanism of action of rituximab is presented in Figure 1. Genetic variations in FcγRIIIa may affect its efficacy. Although generally well tolerated, RTX can cause infusion-related reactions (IRRs), especially during the first infusion, that range from mild to severe, including rare fatal cases. These reactions are also observed with other immune-targeting therapies. Further research is needed to predict and manage these risks. In RA, RTX is recommended for patients who are unresponsive to other biologics and is effective, especially in combination with methotrexate. This knowledge has led to the development of next-generation anti-CD20 therapies like obinutuzumab, which enhances ADCC and reduces CDC, reflecting an improved efficacy [24,25,26,27,28,29,30,31,32,33,34,35].

Rituximab may be effective for rare, aggressive cancers such as sarcomas and is considered a safer biologic disease-modifying anti-rheumatic drug (bDMARD) for those with a history of malignancies. Regular monitoring is crucial to preventing the recurrence of both inflammatory joint conditions and cancer, particularly in rare malignancies like intracranial chondrosarcoma or osteochondroma [36,37,38]. It is also effective in treating RA-related large granular lymphocyte leukemia (LGLL) [39]. In refractory RA, RTX combined with cyclophosphamide or methotrexate shows promise, although further controlled studies are needed to establish its optimal dosage, response rates, long-term effectiveness, and place in RA treatment [40]. A case report of RA with cold agglutinin syndrome (CAS) and immune thrombocytopenia (ITP) showed that full remission was achieved with RTX, further supporting its potential effectiveness in autoimmune complications [41].

RTX is an effective and long-lasting option for patients with RA, particularly those who do not respond to TNF inhibitors, although its superiority over anti-TNF therapies is still debated. There is no consensus on the optimal dosage, but lower doses may benefit patients who are at risk of infection or those who are unable to tolerate standard doses. Caution is needed for patients with chronic infections like hepatitis B, and monitoring is advised for rare paradoxical reactions. Predicting the response of patients to RTX is in its early stages, with genetic markers showing potential for optimization [42,43]. Rituximab has also demonstrated effectiveness in immune-inflammatory disorders with and without B-cell malignancies, in combination with a proposed biomarker panel to assess B-cell depletion and categorize memory B cells [44]. Variations in patient responses are influenced by B-cell turnover [45], and a link between the RTX levels, CD4+ count, and DAS28 in patients with RA has been observed, with CD4+ count decrease correlating with better clinical outcomes [46].

RTX is cost-effective compared to other DMARDs but not as a third-line treatment after biologic therapies fail. Further evidence tailored to specific settings, particularly low-income contexts, is needed [47]. In managing RA-associated interstitial lung disease (ILD), RTX can help improve the lung function, even in those with a usual interstitial pneumonia (UIP) pattern [48,49]. For RA-related pericarditis, RTX has emerged as a promising treatment, given limited success with conventional treatments [50]. RTX therapy is linked to infusion reactions such as fever, chills, and allergic reactions, which are typically more severe during the first infusion and more common in patients with hematologic cancers [51]. Rare adverse effects include necrotizing scleritis, macular edema, and vision loss, as well as progressive multifocal leukoencephalopathy [52,53].

Prolonged RTX therapy may lead to hypogammaglobulinemia (HGG) and requires careful monitoring for infections [54,55]. However, RTX therapy does not pose a higher infection risk than other treatments [56]. In primary Sjögren’s syndrome (pSS), combining RTX with belimumab showed greater B-cell depletion in the salivary glands compared to monotherapy, potentially improving clinical outcomes [57]. RTX combined with methotrexate (MTX) is effective and safe for patients with RA [58], and while an indirect comparison between RTX and tocilizumab suggests that tocilizumab is superior for ACR20 achievement, RTX remains an option for non-responders as a fourth-line therapy [59].

A gradual reduction in RTX after initial improvement in patients with long-standing RA with comorbidities resulted in low relapse rates and fewer serious adverse events [60]. The RNA sequencing of RA synovial tissue has shown a stronger correlation with treatment responses than traditional histopathology. Patients with low or absent B-cell lineage signatures tend to respond better to tocilizumab than RTX. As the RA disease subtypes vary in their clinical presentation, integrating molecular pathology signatures into treatment decisions can optimize therapies and guide the development of new treatments for resistant disease [61,62]. The PRAIRI study demonstrated that B-cell depletion with RTX delayed the onset of RA in patients with arthralgia, underlining he critical role of B cells in RA development [63].

Rituximab’s patent expired in 2015 in both Europe and the U.S., paving the way for the approval of CT-P10, the first biosimilar of RTX for RA. CT-P10 provides a cost-effective alternative to the original RTX, offering similar efficacy and safety. Clinical trials have shown no significant differences in treatment responses between CT-P10 and RTX, even with switching [64]. Systematic reviews and meta-analyses support the equivalence of rituximab’s biosimilar to the original drug in treating RA and non-Hodgkin lymphoma (NHL), reinforcing its clinical utility [65]. The approval of CT-P10 (Truxima) and another biosimilar, GP2013 (Rixathon), demonstrates their comparable effectiveness and safety, with CT-P10 having been approved in multiple countries [66].

Rituximab has demonstrated significant efficacy in various lymphoid malignancies. In diffuse large B-cell lymphoma (DLBCL), the addition of rituximab to cyclophosphamide, doxorubicin, vincristine, and prednisone (CHOP) chemotherapy significantly improved clinical outcomes. According to [67], patients treated with rituximab plus CHOP (R-CHOP) had a 10-year overall survival (OS) rate of 43.5% compared to the 27.6% observed in the CHOP-only group, and the progression-free survival (PFS) at 10 years was 36.5% versus 20.1%, respectively. In follicular lymphoma (FL), the PRIMA study reported by Jurczak et al. showed that 2-year maintenance therapy with rituximab extended the median progression-free survival (PFS), when used in combination with cyclophosphamide, vincristine, and prednisone (CVP), from 4.1 years to 10.5 years, with a hazard ratio (HR) of 0.55 (95% CI: 0.44–0.68; *p* < 0.0001) [68]. In chronic lymphocytic leukemia (CLL), Chihara et al. (2020) [68] reported that combining rituximab with fludarabine and cyclophosphamide (FC) resulted in a complete remission (CR) rate of 97% versus the 24% obtained for cladribine alone, and 94% of patients remained minimal residual disease (MRD)-free at 96 months in the concurrent arm, compared to only 12% in the delayed group (*p* < 0.0001). The efficacy of rituximab is also evident in autoimmune diseases. In the EVER-ILD randomized trial [69], the authors found that the combination of rituximab with mycophenolate mofetil produced a mean increase in the forced vital capacity (FVC) of +1.60% (standard error [SE] 1.13) after 24 weeks, compared to the decrease of −2.01% (SE 1.17) that was obtained in the placebo + Mycophenolate Mofetil (MMF) group, resulting in a statistically significant between-group difference of 3.60% (95% CI: 0.41–6.80; *p* = 0.0273). Moreover, the progression-free survival was significantly better in the rituximab + MMF group (HR 0.47; 95% CI: 0.23–0.96; *p* = 0.03) [70].

A summary of the clinical efficacy of rituximab in selected indications is presented in Table 1.

Despite these successes, the clinical use of rituximab faces notable limitations. In the [71], the authors documented that infusion-related reactions occurred in up to 77% of patients during their first dose, with the majority being mild to moderate, although rare anaphylactic reactions have been observed. Late-onset neutropenia affected 10–25% of patients, with grade 3–4 severity being reported particularly in patients who were receiving chemoimmunotherapy. Hepatitis B virus (HBV) reactivation occurred in fewer than 5% of the screened populations but could lead to fulminant hepatitis if antiviral prophylaxis was not administered. Progressive multifocal leukoencephalopathy (PML), a rare but often fatal complication, occurred in <0.1% of the treated individuals. Hypogammaglobulinemia was seen in approximately 15–30% of long-term rituximab users, potentially leading to recurrent infections, especially in patients with autoimmune indications.

A summary of the above adverse effects associated with rituximab across disease contexts is shown in Table 2.

The high cost of the originator of rituximab previously limited its accessibility. However, the approval of the biosimilars CT-P10 and GP2013 has broadened its therapeutic use. No significant differences in efficacy, pharmacokinetics, or immunogenicity were noted between the biosimilars and the reference product, facilitating substantial health and economic benefits [67,68]. Therapeutic challenges also include determining the optimal dosing schedules and treatment duration of these drugs, particularly for maintenance therapy in indolent lymphomas. Furthermore, primary and secondary resistance mechanisms, such as CD20 downregulation or polymorphisms in Fc receptors, may affect the treatment outcomes and warrant biomarker-guided therapeutic strategies. Based on the current body of evidence, rituximab remains a cornerstone therapy for B-cell malignancies and selected autoimmune diseases. Future research should prioritize head-to-head comparisons of biosimilars, the evaluation of combination regimens in refractory cases, and the development of predictive biomarkers to optimize patient selection. Moreover, long-term pharmacovigilance is crucial to the monitoring of delayed adverse effects and secondary malignancies.

## 3. Targeted Synthetic Disease-Modifying Antirheumatic Drugs (tsDMARDS)

Over the past few years, multiple studies have underscored the significant role of signaling pathway dysfunction in PsA development, particularly those that involved the PDE4 enzyme and the Janus kinase (JAK)–signal transducer and activator of transcription (STAT) pathway. These pathways are key targets for a class of medications known as targeted synthetic DMARDs (tsDMARDs). The emergence of tsDMARDs has expanded the treatment options for PsA and other inflammatory diseases. Among these, the PDE4 inhibitor apremilast and JAK/STAT pathway inhibitors—such as tofacitinib, filgotinib, baricitinib, and upadacitinib—are classified as oral small molecules (OSMs). These low-molecular-weight drugs work by reducing the production of pro-inflammatory cytokines while regulating the release of anti-inflammatory mediators. Oral small-molecule targeted DMARDs function in a manner that is similar to that of biologic DMARDs, primarily by inhibiting JAK, a group of intracellular proteins involved in the JAK/STAT signaling pathway. Blocking JAK influences downstream biological processes, ultimately helping to regulate immune cell activity and inflammation. While generally well tolerated, JAK inhibitors may lead to side effects such as serious infections, kidney damage, anemia, liver abnormalities, and blood clots. These targeted DMARDs are typically prescribed for patients who do not respond to biologic DMARDs or those who are unable to undergo injections or infusions. However, ongoing research is investigating their potential use earlier in the treatment of RA. They can be administered alone or in combination with traditional DMARDs but should not be used alongside biologic DMARDs [72,73].

### 3.1. PDE4 Inhibitor—Apremilast

**Figure 2:**
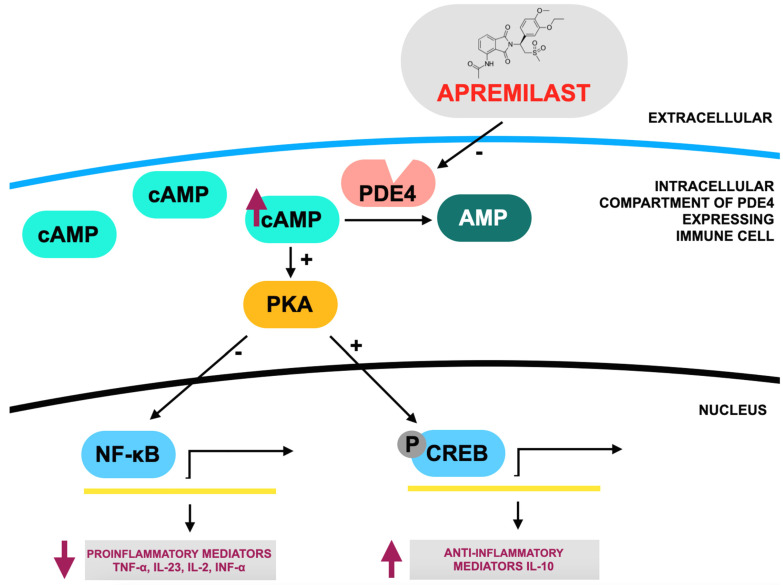
Mode of action of apremilast according to [101], where: PDE4—phosphodiesterase 4; PKA—protein kinase A; cAMP—cyclic adenosine monophosphate; CREB—cAMP response element-binding protein; NF-κB—nuclear factor kappa B. Apremilast acts as a competitive inhibitor of phosphodiesterase 4 (PDE4), resulting in an increase in intracellular cyclic adenosine monophosphate (cAMP) levels. Elevated cAMP triggers downstream modifications in gene expression, reducing the production of pro-inflammatory mediators such as TNF-α, IL-23, IL-2, and IFN-α while enhancing the expression of anti-inflammatory mediators like IL-10.

The anti-inflammatory effects of PDE inhibitors have been recognized since the 1970s, with selective PDE4 inhibitors like apremilast proving effective in treating inflammatory and autoimmune diseases. Apremilast, an oral PDE4 inhibitor, is approved for moderate to severe psoriatic arthritis (PsA) in the U.S. and Europe, with its efficacy having been confirmed in phase III trials. It increases immune-regulatory cAMP levels, suppressing inflammatory cytokines like TNF-α and IL-23, while reducing IL-12, CXCL9, CXCL10, CCL4, and MMP-3, which contribute to cartilage damage. Unlike biologics, it modulates immune pathways earlier without suppressing IL-10 [74,75,76,77,78,79,80,81,82,83]. The mechanism of action of apremilast is presented in Figure 2.

In phase III ESTEEM trials, 30 mg twice-daily apremilast significantly improved moderate to severe plaque psoriasis, including hard-to-treat areas, with symptom relief being observed as early as week 2. Phase III PALACE trials confirmed its benefits for PsA, including enthesitis, dactylitis, and fatigue, with sustained effects for up to 208 weeks. The phase IIIb ACTIVE trial showed early effectiveness, with significant ACR20 improvements by week 2. Apremilast is well tolerated, requires no lab monitoring, and is particularly beneficial for patients with comorbidities like infections or neoplasms [84,85,86,87,88]. As a tsDMARD, apremilast modulates inflammatory mediators and is effective in PsA, including cases that are unresponsive to anti-TNF therapy. While a small study suggested potential benefits in ankylosing spondylitis, the larger POSTURE trial showed no significant improvement. Phase II and III trials confirmed its effectiveness in peripheral PsA for patients failing conventional DMARDs [89]. FDA-approved for psoriasis, PsA, and oral ulcers in Behçet’s disease, apremilast is also used off-label for conditions like atopic dermatitis, lichen planus, and hidradenitis suppurativa. Despite its clinical benefits, NICE does not recommend it for patients with active PsA who are unresponsive to DMARDs [90,91]. It shows promise as a standalone therapy for oligoarticular PsA when csDMARDs fail [92]. Psoriasis, linked to multiple comorbidities, is managed with topical treatments for mild cases and systemic therapies for moderate to severe disease. Apremilast, alongside methotrexate, acitretin, and cyclosporine, remains an option for patients who prefer oral therapy over biologics [93,94,95].

PsA manifests in five clinical forms, requiring tailored treatment strategies. NSAIDs are used for mild cases, while DMARDs address moderate to severe disease. Apremilast (Otezla), approved in 2014 for PsA and later for plaque psoriasis, inhibits pro-inflammatory cytokines and offers an oral alternative to biologics. Phase III trials (PALACE, ACTIVE) confirmed its efficacy, although though it is less potent than biologics, particularly in patients who failed previous treatments. It is recommended for those who are unresponsive to DMARDs and unsuitable for biologics, and exhibits mild gastrointestinal side effects [96,97,98,99,100,101,102]. Apremilast shows potential in severe palmoplantar pustulosis (PPP), but further studies are needed [103]. The GRAPPA guidelines recommend it for patients with peripheral PsA who are unresponsive to csDMARDs, given its safety in patients who are at high risk of infections, cancer, or tuberculosis [72]. Combination therapy with biologics may be beneficial for patients with a reduced biologic response [104]. Its long-term safety aligns with that seen in clinical trial data [105].

The ESTEEM 1/2 and PALACE 3 trials suggest metabolic benefits, including weight loss and improved insulin sensitivity, of csDMARDs. Real-world studies confirm PASI and quality-of-life improvements, making apremilast a second-line treatment for obese psoriasis patients and a third-line option for those with ulcerative colitis [106,107]. The APROACH study highlights early and lasting improvements in PsA symptoms with apremilast. In Greece, patients who are biologic-naïve showed strong treatment persistence and safety [108]. Apremilast maintains a positive benefit–risk balance for PsA, psoriasis, and Behçet’s syndrome, with a low incidence of adverse events (<1%) at 30 mg [109]. A 5-year PALACE 4 analysis confirmed sustained PsA improvement and consistent safety [110]. However, a large retrospective study reported low treatment persistence, mainly due to inefficacy in treating joint symptoms, despite no demographic or clinical factors affecting its discontinuation [111].

Finnish clinical practice places apremilast between csDMARDs and biologics, with persistence rates comparable to international data, favoring older patients with comorbidities [112]. In Spain, patients with biologic-naïve PsA who were on apremilast showed reduced joint symptoms and an improved quality of life, with two-thirds remaining on treatment at 12 months [113]. Canadian real-world data confirm ongoing efficacy and patient satisfaction [114]. Meta-analysis shows that apremilast significantly improves HAQ-DI, ACR20, ACR50, and ACR70 scores, enhancing functionality while causing mild gastrointestinal side effects, with serious adverse events comparable to placebo [115]. The FOREMOST trial, the first RCT on early oligoarticular PsA, demonstrated superior disease control and a superior MDA joints response at 16 weeks, warranting further research [116].

The clinical efficacy of apremilast has been extensively evaluated in randomized clinical trials and real-world studies, and its application has also been expanded to several inflammatory skin diseases beyond psoriasis. One of the most widely used clinical tools to assess disease severity and response to treatment in psoriasis is the Psoriasis Area and Severity Index (PASI), which combines the severity of lesions and the extent of affected body surface area into a single score ranging from 0 (no disease) to 72 (maximal disease) [90]. Treatment responses are commonly reported as PASI-50, PASI-75, or PASI-100, corresponding to ≥50%, ≥75%, and 100% improvement from the baseline PASI score, respectively. In the phase III ESTEEM 1 trial, 33.1% of patients treated with 30 mg of apremilast twice daily achieved a 75% reduction in the Psoriasis Area and Severity Index (PASI-75) at week 16, compared to the 5.3% that was observed in the placebo group (*p* < 0.0001). At week 52, 61% of patients rerandomized to apremilast maintained a PASI-75 response, and the mean PASI improvement was sustained between −88% and −81% from baseline [117].

Similarly, the ESTEEM 2 trial showed a PASI-75 response at week 16 in 28.8% of patients versus 5.8% in the placebo group (*p* < 0.001), with a PASI-50 response being maintained in 80% of patients at week 52 [118]. Real-world data also support apremilast’s efficacy. In a Greek observational cohort, 59.3% of patients reached PASI-75 by week 16, with an additional 11.1% achieving a PASI-50 combined with a Dermatology Life Quality Index (DLQI) ≤ 5. Complete skin clearance (PASI-100) was achieved in 18.5% of patients. Drug discontinuation occurred in 28% of cases—12% due to adverse effects (mainly gastrointestinal symptoms) and 16% due to insufficient efficacy [119]. Beyond psoriasis, apremilast has shown benefits in other inflammatory conditions. In a phase III trial involving 207 patients with Behçet’s disease and recurrent oral ulcers, 30 mg of apremilast twice daily significantly reduced the ulcer count and area compared to placebo (AUC 129.5 vs. 222.1; *p* < 0.0001) and improved the quality of life (−4.3 vs. −1.2; *p* = 0.0003) of patients [120]. In atopic dermatitis, a small open-label pilot study showed reductions in pruritus and Eczema Area Severity Index (EASI) scores after 12 weeks of apremilast therapy. In patients with cutaneous sarcoidosis, case reports describe reduced granulomatous inflammation and improved lesion resolution. Similarly, in hidradenitis suppurativa, reductions in inflammatory nodules and improvements in pain and drainage were observed after 16 weeks of treatment [90]. To aid interpretation of the results discussed above, it is important to define “the area under the curve” (AUC), often used in trials such as those for Behçet’s disease, which reflects the cumulative intensity or burden of a symptom—such as oral ulcers—over time. A summary of the clinical efficacy of apremilast in selected indications is presented in Table 3.

To provide an overview of apremilast’s tolerability profile, a summary of the adverse effects of apremilast is presented in Table 4. This table compiles the most commonly reported adverse events from clinical trials and real-world studies, with an emphasis on their frequency and clinical relevance. Gastrointestinal disturbances such as diarrhea and nausea are among the most frequent, but are typically mild and transient [117]. Other common side effects include upper respiratory tract infections, nasopharyngitis, headache, and moderate weight loss [117,119].

While apremilast offers a favorable safety profile and oral administration convenience, several challenges remain in its clinical use. Its relatively modest efficacy compared to biologics, particularly in achieving complete skin clearance (PASI-100), can limit its utility in patients with severe disease. Moreover, gastrointestinal side effects—though typically mild—are among the most common reasons for treatment discontinuation in both trials and real-world settings [117,119]. Long-term adherence may also be influenced by its delayed onset of action and patients’ expectations. Unlike biologics, apremilast lacks validated biomarkers for predicting the treatment response, which complicates personalized treatment decisions [90]. As a small-molecule drug, apremilast does not yet face biosimilar competition; however, its cost-effectiveness remains a consideration, especially in healthcare systems with limited resources. Future research should focus on identifying predictive markers of response, optimizing patient selection, and exploring combination approaches to enhance its efficacy in refractory or severe inflammatory dermatoses. Additionally, post-marketing surveillance is essential to further characterize its long-term safety in broader patient populations [90,117].

### 3.2. JAK1 Inhibitor—Upadacitinib

**Figure 3:**
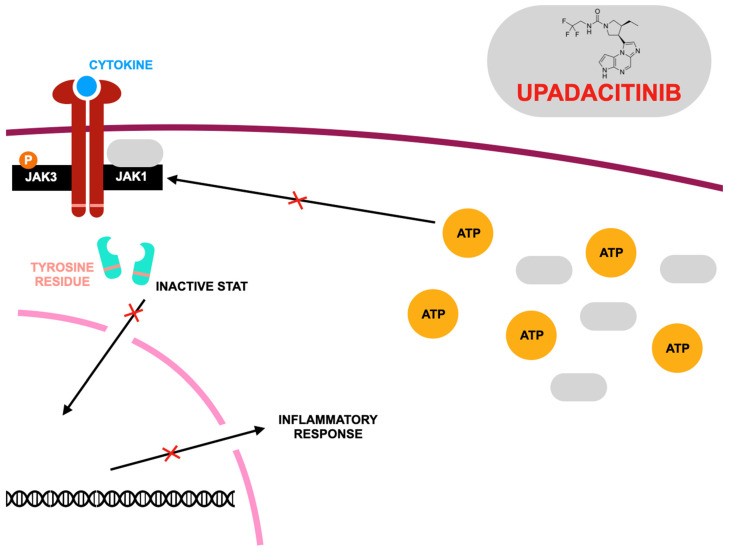
Mode of action of upadacitinib according to [135], where: JAK1,3—Janus family tyrosine kinases; STAT—signal transducers and activators of transcription; ATP—adenosine triphosphate. Upadacitinib acts as a competitive inhibitor of adenosine triphosphate (ATP) at Janus kinase (JAK) binding sites, preventing ATP from attaching and thereby blocking nucleotide binding. This inhibition disrupts kinase activity and stops the phosphorylation of downstream signaling molecules. As a result, STAT dimers fail to form, preventing their movement into the nucleus and their interaction with gene promoters. Since different cytokine receptors utilize distinct JAK kinase combinations, these variations play a crucial role in determining therapeutic strategies for targeting JAKs in various diseases. Upadacitinib primarily inhibits JAK1 with high potency, while its effects on other isoforms—JAK2, JAK3, and TYK2 (tyrosine kinase 2)—are comparatively weaker.

Cytokines play a central role in chronic immune-mediated diseases like atopic dermatitis, rheumatoid arthritis (RA), and psoriatic arthritis and gastrointestinal conditions such as ulcerative colitis and Crohn’s disease. While biologic drugs target extracellular cytokines, newer treatments focus on disrupting intracellular signaling, particularly through the JAK-STAT pathway, which is vital in autoimmune diseases. Four JAK enzymes—JAK1, JAK2, JAK3, and TYK2—are involved in cytokine signaling, with recent advances leading to the development of pan-JAK and selective-JAK inhibitors such as tofacitinib, baricitinib, and upadacitinib, the first JAK inhibitors to be commercially available. Upadacitinib, a selective JAK1 inhibitor, has demonstrated a strong benefit–risk profile across various conditions, including rheumatic diseases, improving patients’ symptoms, pain, and function while providing better disease control [121,122,123,124,125,126,127]. The mechanism of action of upadacitinib is presented in Figure 3.

Approved by the FDA, EMA, and other regulatory agencies, upadacitinib (Rinvoq) treats chronic inflammatory diseases such as RA, psoriatic arthritis, and ulcerative colitis by inhibiting JAK1, disrupting cytokine signaling. It is especially effective in patients with RA who have not responded to methotrexate (MTX) or other csDMARDs and is available in 15 mg extended-release tablets, either for use as monotherapy or in combination with other DMARDs [128,129,130].

Upadacitinib also shows promise for severe nail psoriasis [131]. The SELECT phase III program confirmed its efficacy in RA, with 15 mg of upadacitinib daily being approved as both monotherapy and in combination with csDMARDs. In Japan, 7.5 mg and 15 mg doses were approved, although 7.5 mg did not show advantages in reducing adverse events or improving remission rates compared to the higher dose. Overall, it remains a strong treatment for patients with insufficient responses to conventional or biologic therapies [132]. In clinical trials for atopic dermatitis (AD), upadacitinib outperformed dupilumab after 16 weeks, showing faster and more significant improvements in skin clearance and itch relief, making it a promising AD treatment [133]. It also showed effectiveness and a favorable safety profile in treating moderate-to-severe ulcerative colitis [134]. A systematic review found that upadacitinib improves remission rates and endoscopic outcomes in Crohn’s disease, especially for patients who are unresponsive to conventional treatments, although safety concerns such as herpes zoster and intestinal perforations remain [135,136]. In patients with experimental autoimmune uveitis (EAU), upadacitinib demonstrated therapeutic effectiveness, suggesting further potential for treating uveitis [137].

The SELECT-MONOTHERAPY trial showed that upadacitinib monotherapy led to significant improvements in disease outcomes, offering an option for patients who are intolerant to MTX or those who prefer treatments without concurrent csDMARDs [138]. In patients with RA who are unresponsive to biologic DMARDs, upadacitinib outperformed abatacept in improving DAS28-CRP scores and achieving remission, although it was linked to more serious adverse events, warranting further long-term studies [139]. A study also showed that 15 mg and 30 mg of upadacitinib were more effective than placebo in treating psoriatic arthritis, with the 30 mg dose being more effective than adalimumab [124]. Additionally, 15 mg and 30 mg of upadacitinib demonstrated better results in alleviating PsA symptoms compared to placebo [140].

Network meta-analyses have highlighted that JAK1 inhibitors, including upadacitinib, showed greater efficacy than placebo for moderate-to-severe atopic dermatitis. Upadacitinib 30 mg was particularly effective, providing superior investigator global assessment (IGA) and EASI responses compared to other treatments, although more efforts are needed to minimize treatment-emergent adverse events (TEAEs) [141]. Long-term data suggest that upadacitinib is well tolerated across multiple indications, with no new safety concerns [142]. A separate study evaluated the clinical effectiveness and cost-effectiveness of upadacitinib for atopic dermatitis, indicating that 30 mg of upadacitinib may be more effective than other treatments, and that it has potential for use as a first-line therapy [143].

The therapeutic potential of upadacitinib has been evaluated across a broad range of immune-mediated inflammatory diseases. This section provides a summary of key clinical efficacy data, safety findings, and real-world considerations from randomized trials and observational studies involving patients with conditions such as psoriatic arthritis, Crohn’s disease, and atopic dermatitis. Particular attention is given to validated endpoints including the American College of Rheumatology (ACR) response criteria, clinical remission, the endoscopic response, and EASI scores, which form the basis of efficacy assessment. In psoriatic arthritis, the SELECT-PsA 1 phase III trial showed that upadacitinib was superior to placebo and, at a 30 mg dose, also superior to adalimumab. At week 12, 78.5% of patients receiving 30 mg once daily achieved an ACR20 response, compared to the 70.6% that was observed in the 15 mg group, the 36.2% observed in the placebo group, and the 65.0% obtained with adalimumab. The difference versus adalimumab was +13.5% for 30 mg (95% CI, 7.5–19.4) [130]. 

In moderate-to-severe Crohn’s disease, the U-EXCEL and U-EXCEED induction trials demonstrated clinical remission at week 12 in 49.5% and 38.9% of patients treated with 45 mg of upadacitinib daily, compared to 29.1% and 21.1% in the placebo groups, respectively. The endoscopic response was also significantly higher with upadacitinib (45.5% and 34.6% vs. 13.1% and 3.5%; all *p* < 0.001). In the U-ENDURE maintenance trial, 47.6% of patients on 30 mg daily maintained remission at week 52 compared to the 15.1% obtained with placebo [136]. In moderate-to-severe atopic dermatitis, the phase IIIb HEADS UP study directly compared 30 mg of upadacitinib daily with dupilumab. The EASI is a validated scoring system used to assess the severity and extent of atopic dermatitis, combining evaluations of the redness, thickness, scratching, and lichenification across body regions. At week 16, 72.4% of patients on upadacitinib achieved EASI-75 versus 62.6% on dupilumab (*p* = 0.007), and 28.4% versus 7.9% achieved EASI-100 (*p* < 0.001). The improvements in pruritus were also significantly faster and greater in the upadacitinib group from week 1 onward [133]. A summary of the clinical efficacy of upadacitinib in selected indications is presented in Table 5.

To provide a comprehensive overview of upadacitinib’s tolerability profile, the most frequently reported adverse effects are presented in Table 6. These data are derived from phase III clinical trials [130,133,136] and reflect adverse events that occurred with notable frequency across different disease populations. Particular attention is given to events such as acne, infections, laboratory abnormalities, and herpes zoster, the frequencies of which vary depending on the indication, dose, and patient-specific factors. Understanding the safety profile is critical for treatment selection and monitoring in clinical practice.

Upadacitinib has shown promising outcomes in difficult-to-treat rheumatoid arthritis (D2T-RA), particularly in patients who are unresponsive to multiple biologic or targeted synthetic DMARDs [144,145]. In the SELECT-BEYOND trial, 65% of patients achieved ACR20 at week 12 compared to the 28% obtained with placebo [130]. Real-world data from the ELECTRA-i study reported ~80% 12-month treatment retention, with significant reductions in disease activity measured by the DAS28-CRP (Disease Activity Score in 28 joints with C-reactive protein) and CDAI (Clinical Disease Activity Index). A substantial portion of patients reached low disease activity (DAS28-CRP < 3.2 or CDAI ≤ 10) and remission by month 12 [145].

Despite these benefits, safety concerns remain. Cardiovascular comorbidities are particularly relevant in this context, as patients with RA are at an increased baseline risk of atherosclerosis and thrombotic events. The EMA and FDA have warned of increased risk of MACEs (major adverse cardiovascular events), VTE (venous thromboembolism), and malignancy in older or high-risk individuals [146,147]. Upadacitinib may further exacerbate this risk in predisposed patients, highlighting the need for baseline cardiovascular assessment and ongoing monitoring. Although its overall safety appears comparable to that of adalimumab, its CYP3A4-mediated interactions and limited long-term data warrant caution. Patient-specific risk stratification and further real-world research are needed to define its role in high-risk RA populations.

To facilitate the direct comparison of rituximab, apremilast, and upadacitinib, their main pharmacologic properties, clinical efficacy, safety profiles, and unique considerations for different indications are summarized in Table 7.

## 4. Conclusions

Arthritis is a multifaceted and often disabling autoimmune condition that not only damages the joints but also contributes to systemic complications involving various organ systems. Its underlying causes are rooted in the dynamic interplay of genetic predisposition, epigenetic modifications, and environmental triggers, all of which contribute to persistent inflammation, immune system imbalance, and progressive tissue destruction. Despite substantial progress in the development of disease-modifying antirheumatic drugs, a significant proportion of patients continue to experience inadequate therapeutic responses. This highlights the ongoing need to deepen our understanding of the disease’s cellular signaling pathways and molecular drivers.

Recent therapeutic advancements have introduced new classes of DMARDs, including biologics, biosimilars, and targeted synthetic agents, which have broadened treatment possibilities and provided renewed hope for individuals with treatment-resistant rheumatoid arthritis (RA). Nevertheless, the clinical challenge remains to refine therapeutic approaches, reduce adverse effects, and address the disease’s comorbid burden. Emerging strategies in personalized medicine, novel drug development, and integrated multidisciplinary care hold promise for improving the outcomes for and enhancing the quality of life of individuals living with arthritis. The development of next-generation DMARDs—such as biologic agents, biosimilar products, and targeted synthetic therapies—has significantly changed the treatment landscape for chronic autoimmune and inflammatory disorders. Among these, rituximab, apremilast, and upadacitinib represent three pharmacologically distinct yet complementary agents that exemplify innovation in immune modulation. Rituximab is a monoclonal antibody that selectively targets CD20-expressing B cells, offering a substantial benefit in both autoimmune diseases and B-cell malignancies, especially in patients who have not responded to TNF inhibitors. Biosimilar versions like CT-P10 and GP2013 have expanded global access to this therapy by reducing costs without compromising efficacy or safety.

Apremilast, an oral phosphodiesterase 4 (PDE4) inhibitor, is notable for its well-tolerated safety profile and is particularly useful for patients with multiple comorbidities or those seeking alternatives to injectable biologics. Its anti-inflammatory effects span several conditions, including psoriatic arthritis, making it a valuable non-biologic therapeutic option. Upadacitinib, a highly selective JAK1 inhibitor, has shown a rapid onset of action and notable effectiveness across various autoimmune disorders, including RA and psoriatic arthritis. Its superiority observed in clinical trials compared to some established therapies has made it a preferred choice in moderate-to-severe disease cases, particularly where fast symptom relief is crucial. Clinically, these three agents offer flexible treatment strategies tailored to the individual’s disease severity, coexisting conditions, and personal treatment preferences.

The diverse mechanisms of action, administration methods, and safety considerations of these drugs enable clinicians to make nuanced decisions—whether initiating therapy, escalating treatment in resistant cases, or managing systemic involvement beyond the joints. Moreover, their applications span multiple specialties, reinforcing the importance of collaborative care models involving rheumatology, dermatology, gastroenterology, and oncology. In practice, rituximab biosimilars should be viewed as cost-effective options for managing B-cell–mediated diseases, especially in cases with overlapping autoimmune and oncologic features. Apremilast continues to be an important oral alternative for patients who require close safety monitoring or who wish to avoid injections. Upadacitinib, due to its potent immunosuppressive effects, is particularly valuable in patients who need swift disease control, although clinicians must remain vigilant regarding cardiovascular and thrombotic risks.

Looking ahead, future research should focus on refining the criteria for selecting the most appropriate therapy for individual patients, potentially through biomarker-guided approaches. Investigating the efficacy of combination treatments in refractory disease, optimizing long-term safety monitoring, and determining effective tapering protocols will be essential. Comparative studies between biosimilars, tsDMARDs, and emerging small molecules will also be critical in guiding future clinical decision-making and updates to treatment guidelines. Rituximab, apremilast, and upadacitinib embody the progression and diversification of the modern DMARD arsenal. Each contributes uniquely to the management of inflammatory and autoimmune disorders, enhancing the capacity for personalized care. Their growing use reflects the broader trend toward precision medicine, adaptive treatment models, and cross-specialty integration—cornerstones of future chronic disease management. As the field evolves, these agents will continue to play a central role in bridging gaps in treatment efficacy, safety, accessibility, and patient-centered care.

## Figures and Tables

**Table 1:** Clinical efficacy of rituximab in selected indications based on [68,69,70].

Disease	Treatment Regimen	Key Clinical Outcomes	Ref.
Diffuse large B-cell lymphoma (DLBCL)	RTX + CHOP	10-year overall survival: 43.5% vs. 27.6%; PFS: 36.5% vs. 20.1%	[68]
Follicular lymphoma (FL)	RTX + CVP (+ Maintenance)	Median PFS: 10.5 vs. 4.1 years; HR: 0.55 (95% CI: 0.44–0.68); *p* < 0.0001	[68]
Chronic lymphocytic leukemia (CLL)	RTX + Fludarabine + Cyclophosphamide	CR rate: 97% vs. 24%; MRD-free at 96 months: 94% vs. 12%	[69]
Interstitial Lung Disease (EVER-ILD)	RTX + Mycophenolate Mofetil	ΔFVC: +1.60% vs. −2.01%; PFS HR: 0.47 (95% CI: 0.23–0.96); *p* = 0.03	[70]

PFS—progression-free survival; OS—overall survival; CR—complete response; HR—hazard ratio; CI—confidence interval; MRD—minimal residual disease; FVC—forced vital capacity; CHOP—cyclophosphamide, doxorubicin, vincristine, prednisone; CVP—cyclophosphamide, vincristine, prednisone.

**Table 2:** Overview of selected rituximab-associated adverse events based on [71].

Adverse Event	Frequency	Severity	Risk Factors	Clinical Context
Infusion-related reactions	Up to 77% (first dose)	Mild to severe; rare anaphylaxis	First cycle, infusion speed	Across all indications
Late-onset neutropenia	10–25%	Grade 3–4 (notable in combination therapy)	Chemoimmunotherapy	Hematologic malignancies
Hepatitis B reactivation	<5%	May be fulminant without prophylaxis	Chronic HBV, lack of screening	Immunocompromised patients
Progressive multifocal leukoencephalopathy	<0.1%	Often fatal	JC virus reactivation	Long-term treatment
Hypogammaglobulinemia	15–30% (long-term)	Mild to moderate; risk of infections	Cumulative exposure	Autoimmune disorders

HBV—hepatitis B virus; JC—John Cunningham virus.

**Table 3:** Clinical efficacy of apremilast in selected indications based on [90,117,118,119,120].

Disease	Treatment Regimen	Key Clinical Outcomes	Ref.
Moderate-to-severe plaque psoriasis (ESTEEM 1)	Apremilast 30 mg BID	PASI-75 at week 16: 33.1% vs. 5.3% (placebo); PASI-75 at week 52: 61%	[117]
Moderate-to-severe plaque psoriasis (ESTEEM 2)	Apremilast 30 mg BID	PASI-75 at week 16: 28.8% vs. 5.8%; PASI-50 at week 52: 80%	[118]
Moderate psoriasis (real-world)	Apremilast 30 mg BID	PASI-75 at week 16: 59.3%; PASI-100: 18.5%; discontinuation rate: 28%	[119]
Behçet’s disease	Apremilast 30 mg BID	AUC for oral ulcers: 129.5 vs. 222.1; QoL score change: −4.3 vs. −1.2	[120]
Atopic dermatitis, cutaneous sarcoidosis, hidradenitis suppurativa (off-label)	Apremilast 30 mg BID	Case-level improvement in lesion severity and pruritus	[90]

PASI—Psoriasis Area and Severity Index; PASI-75—≥ 75% reduction in PASI; QoL—quality of life; BID—twice daily; AUC—area under the curve—often used in trials such as those for Behçet’s disease, reflects the cumulative intensity or burden of a symptom, such as oral ulcers, over time.

**Table 4:** Common adverse events associated with apremilast based on [117,119].

Adverse Event	Frequency	Clinical Impact	Ref.
Diarrhea	17.3%	Mostly mild to moderate; transient	[117]
Nausea	15.7%	Often occurs within first weeks; transient	[117]
URTI	15.5%	Non-severe, self-limiting	[117]
Nasopharyngitis	14.4%	Mild; typically no need for discontinuation	[117]
Headache	6.3–9.0%	Tension-type most frequent	[117]
Weight loss	5–10%	Mild to moderate; may be desired or problematic	[119]

URTI—upper respiratory tract infection.

**Table 5:** Clinical efficacy of upadacitinib in selected indications based on [130,133,136].

Disease	Treatment Dose	Clinical Outcomes	Ref.
Psoriatic arthritis	15/30 mg QD	**ACR20 at week 12: 70.6% (15 mg), 78.5% (30 mg),** vs. 65.0% (adalimumab), 36.2% (placebo)	[130]
Crohn’s disease (induction)	45 mg QD	**Week 12: remission 49.5% (U-EXCEL), 38.9% (U-EXCEED)** vs. 29.1% (U-EXCEL placebo), 21.1% (U-EXCEED placebo)	[136]
Crohn’s disease (maintenance)	30 mg QD	**Week 52 remission: 47.6%** vs. 15.1% (placebo)	[136]
Atopic dermatitis	30 mg QD	**Week 16 EASI-75: 72.4%** vs. 62.6% (dupilumab); **Week 16 EASI-100: 28.4%** vs. 7.9% (dupilumab)	[133]

Bolded values in the table indicate outcomes observed in the upadacitinib treatment; ACR20—≥ 20% improvement in American College of Rheumatology criteria; QD—once daily; EASI—Eczema Area and Severity Index.

**Table 6:** Common adverse events associated with upadacitinib based on [130,133,136].

Adverse Event	Frequency	Clinical Impact	Ref.
Acne	10–15%	Typically mild; more common in atopic dermatitis	[133]
URTI	8–13%	Self-limiting; common across indications	[130,133]
Headache	~10%	Mild to moderate; manageable	[133]
Elevated CPK	2–8%	Asymptomatic in most cases	[133]
Herpes zoster (shingles)	1–3%	Increased risk in elderly or immunocompromised	[136]

CPK—creatine phosphokinase; URTI—upper respiratory tract infection.

**Table 7:** Brief comparative overview of rituximab, apremilast, and upadacitinib based on [130,133,136,144,145,146,147].

Property	Rituximab [67,68,69,70,71]	Apremilast [130,133,136,144,145]	Upadacitinib [130,133,136,144,145,146,147]
Mechanism of Action	Anti-CD20 monoclonal antibody depleting B cells	PDE4 inhibitor increasing intracellular cAMP	Selective JAK1 inhibitor modulating cytokine signaling
Route of Administration	Intravenous infusion	Oral tablet	Oral tablet
Approved Indications	B-cell NHL, CLL, RA, GPA/MPA	Psoriasis, PsA	RA, PsA, AD, CD, UC, AS
Key Clinical Outcomes	R-CHOP in DLBCL: 10-year OS 43.5% vs. 27.6%	PASI-75 at week 16: 33.1% (ESTEEM 1)	ACR20 in PsA: up to 78.5%; CD remission: 49.5% at week 12
Common Adverse Events	Infusion reactions, infections	GI symptoms (nausea, diarrhea), weight loss	Acne, URTI, headache, herpes zoster
Special Considerations	Risk of hepatitis B reactivation, long B-cell recovery time	Slower onset, moderate efficacy	Risk of MACE, VTE; CYP3A4 drug interactions

NHL—non-Hodgkin lymphoma; CLL—chronic lymphocytic leukemia; RA—rheumatoid arthritis; GPA/MPA—granulomatosis with polyangiitis/microscopic polyangiitis; PsA—psoriatic arthritis; AD—atopic dermatitis; CD—Crohn’s disease; UC—ulcerative colitis; OS—overall survival; PASI—Psoriasis Area and Severity Index; ACR—American College of Rheumatology; URTI—upper respiratory tract infection; MACE—major adverse cardiovascular events; VTE—venous thromboembolism; GI—gastrointestinal.

## Data Availability

Data sharing is not applicable to this article.
